# Ensemble deep learning for brain tumor detection

**DOI:** 10.3389/fncom.2022.1005617

**Published:** 2022-09-02

**Authors:** Shtwai Alsubai, Habib Ullah Khan, Abdullah Alqahtani, Mohemmed Sha, Sidra Abbas, Uzma Ghulam Mohammad

**Affiliations:** ^1^College of Computer Engineering and Sciences, Prince Sattam Bin Abdulaziz University, AlKharj, Saudi Arabia; ^2^Department of Accounting and Information Systems, College of Business and Economics, Qatar University, Doha, Qatar; ^3^Department of Computer Science, COMSATS University, Islamabad, Pakistan; ^4^Department of Computer Science and Software Engineering, International Islamic University, Islamabad, Pakistan

**Keywords:** brain tumor, convolutional neural network, long short-term memory, CNN-LSTM, MR images, deep learning

## Abstract

With the quick evolution of medical technology, the era of big data in medicine is quickly approaching. The analysis and mining of these data significantly influence the prediction, monitoring, diagnosis, and treatment of tumor disorders. Since it has a wide range of traits, a low survival rate, and an aggressive nature, brain tumor is regarded as the deadliest and most devastating disease. Misdiagnosed brain tumors lead to inadequate medical treatment, reducing the patient's life chances. Brain tumor detection is highly challenging due to the capacity to distinguish between aberrant and normal tissues. Effective therapy and long-term survival are made possible for the patient by a correct diagnosis. Despite extensive research, there are still certain limitations in detecting brain tumors because of the unusual distribution pattern of the lesions. Finding a region with a small number of lesions can be difficult because small areas tend to look healthy. It directly reduces the classification accuracy, and extracting and choosing informative features is challenging. A significant role is played by automatically classifying early-stage brain tumors utilizing deep and machine learning approaches. This paper proposes a hybrid deep learning model Convolutional Neural Network-Long Short Term Memory (CNN-LSTM) for classifying and predicting brain tumors through Magnetic Resonance Images (MRI). We experiment on an MRI brain image dataset. First, the data is preprocessed efficiently, and then, the Convolutional Neural Network (CNN) is applied to extract the significant features from images. The proposed model predicts the brain tumor with a significant classification accuracy of 99.1%, a precision of 98.8%, recall of 98.9%, and F1-measure of 99.0%.

## 1. Introduction

The era of crucial medical data is approaching as a result of the quick advancements in medical technology. The monitoring, prevention, and diagnosis of infectious tumors depend on the management, collection, and appropriate analysis of data in infectious tumor diagnosis and treatment (Akram et al., 2021; Houssein et al., 2021a; Zhang et al., 2021; Javed et al., 2022; Rizwan et al., 2022b). Cancer is one of the most significant health issues and challenges currently threatening people's lives. The scariest and most dangerous sort of cancer among the various cancers is brain tumors (Ali et al., 2022; Senan et al., 2022). The brain is the most complex organ of the human body that acts through billions of cells and connections called synapses (Alanazi et al., 2022). The human brain is the nervous control center system to control all organs of the human body (Mewada et al., 2020; Rizwan et al., 2022a). Therefore, having an abnormal brain has disastrous effects on people's health. The World Health Organization (WHO) claims that, in 2020, roughly 10 million deaths were recorded because of brain cancer, which is the second-leading death cause globally (Can, 2022). Cancer is considered the most deadly and devastating disease because of its diverse characteristics, less survival rate, and aggressive nature. A misdiagnosed brain tumor results in ineffective medical treatment, which lowers the chances of survival for the patient (Rathod and Khan, 2021; Senan et al., 2022). Different types of tumors are based on their texture, location, and shape, such as the pituitary, glioma, lymphomas, Medulloblastoma, malignant and acoustic neuroma (Alanazi et al., 2022; Kumar et al., 2022).

The major challenge arises in detecting the brain tumor because of the tumor location, type, size, and shape variations (Amin et al., 2021). The brain tumor diagnosis depends on the type and location of the tumor so that the doctors can predict the survival chances of the patients and make decisions for treatment that extent from surgery to radiotherapy and chemotherapy (Kumar et al., 2022). Therefore, identifying and detecting the brain tumor at early stages helps plan the treatment and monitor the condition of patients. It plays a crucial part in enhancing the treatment and ensuring higher chances of survival. Different types of medical imaging and diagnostic techniques are used to obtain information related to tumors. Magnetic Resonance Imaging (MRI) and Computed Tomography (CT) are methods for identifying normal and abnormal growing cells in the brain (Houssein et al., 2021b; Alsaif et al., 2022). The Computed Tomography (CT) scan is used to diagnose the patient using an X-ray and the computer to create the brain images in the axial fragments (Bekhet et al., 2020; Fahmi et al., 2020; Mubashar et al., 2022).

Magnetic resonance imaging creates 2D and 3D images of the body's internal organs without pain or needing surgery (Alanazi et al., 2022). It is one of the most accurate methods for the early identification and diagnosis of cancer. However, determining the type of tumor with MRI is challenging, error-prone, and time-consuming, necessitating radiologists with extensive experience. Due to the variety of tumors, MRI images occasionally have no apparent features that would enable sound decision-making. Consequently the human cannot depends on manual diagnosis (Al-Shoukry et al., 2020; Tiwari et al., 2022). An accurate diagnosis helps the patient to receive adequate treatment and longtime survival. Furthermore, the brain tumor's under-diagnosis is risky because it lowers the effectiveness of treatment and chances of survival. Consequently, using artificial intelligence (AI) techniques has become necessary in the computer-aided diagnostic (CAD) system's ability to diagnose medical images like MRI scans. These techniques help doctors and radiologists make an accurate diagnosis while reducing burden (Al-Shoukry et al., 2020; Gab Allah et al., 2021). The CAD system is divided into different steps, such as a preprocessing step for eliminating the noise from images, a segmentation step used to identify the lesion area and separate it from the rest of the image, a feature extraction step for extracting the significant features that show the tumor and a classification step to classify each image and predicts the abnormality (Lu et al., 2021).

*Motivation:* The ability to distinguish between abnormal and normal tissues makes detecting brain tumors challenging. Brain tumors should be found as soon as possible to allow the patient to recover quickly from the treatment. Patients can receive better care if the brain tumor is initially detected. Early brain tumor detection allows for more incredible characterization of the brain's system structures and their easy identification (Kumar et al., 2022). Despite extensive research, there are still certain limitations in detecting brain tumors because of the unusual distribution pattern of the lesions. Finding a region with a small number of lesions can be difficult because small areas tend to look healthy. Extracting and selecting informative features is also tricky because it decreases classification accuracy (Amin et al., 2022). According to the literature review, numerous machine learning techniques have been employed to categorize MRI images (Rehman et al., 2020; Zhou et al., 2020; Kumar et al., 2021). Recent advances in machine learning have led to the application of numerous deep learning approaches for diagnosing MRI images (Alanazi et al., 2022; Alrashedy et al., 2022; Qureshi et al., 2022; Senan et al., 2022; Zeineldin et al., 2022). The main contributions of the research are given below:

- Propose a hybrid deep learning model based on Convolutional Neural Network-Long Short Term Memory (CNN-LSTM) for classifying and predicting brain tumors through Magnetic Resonance Images (MRI).
- Convolutional neural network removes the noise and extracts the essential features.
- The hybrid deep learning model handles the large capacity of high-dimensional data and improves the model's performance.
- Proposed model provides a promising diagnostic model to diagnose the MRI brain images for brain tumor classification and support the radiologists' and experts' decisions.

The paper is organized into the following sections: Section 2 discusses the literature review of the machine learning and deep learning approaches for identifying brain tumors. Section 3 describes the research design of the proposed work, using the MR image dataset and deep learning models. Section 4 explains the results and discusses them. Sections 5 present the work's conclusion and recommendations for future research.

## 2. Literature review

Many studies are conducted to find brain tumors, and some of the most recent methods are mentioned in this section.

### 2.1. Machine learning techniques

Different machine learning techniques are implemented for various healthcare applications such cognitive health assessment, cervical cancer detection, tumor detection, breast cancer detection (Javed et al., 2020, 2021a,b; Mehmood et al., 2021; Mohiyuddin et al., 2022; Rehman et al., 2022b). The machine learning techniques RF, SVM, AdaBoost1, and RUSBoost, are used in Rehman et al. (2020) to localize the brain tumor on FLAIR scans MRI. These techniques are implemented on the BraTs 2012 dataset for both natural and syntactic images, and the proposed model indicates the result with the best accuracy is 0.98%, sensitivity is 0.92%, specificity is 0.96%, precision is 0.88%, and the dice score is 0.88%. The automatic brain tumor classification system is proposed in Kumar et al. (2021) and the K-nearest neighbor algorithm is used to classify the MRI images as abnormal or normal. The fuzz C-means clustering technique is used for the segmentation of tumor regions. The two datasets MICCAI and BRATS, are used for the experiment and evaluate the results with 96.5% accuracy, 100% sensitivity, and 93% specificity. The manual optimizing model by a machine learning expert is presented in Zhou et al. (2020) and compared with the automated machine learning technique Tree-Based Pipeline optimizing tool for evaluating the model performance. The proposed model was implemented on MRI images of 288 patients, and results showed that the best AUC value is 0.94% and accuracy is 0.88%.

### 2.2. Deep learning techniques

Although deep learning models have been employed in many fields, they still need to be adjusted before they can be used in delicate fields like medical imaging. The GAN architecture is proposed in Alrashedy et al. (2022) and different deep learning models CNN, ResNet152V2, and MobileNetV2 are used to generate and categorize the MRI brain images. The images are created by DCGAN and Vanilla GAN, which are used to train the deep transfer models and evaluate the performance on the test set composed of authentic MRI brain images. The experiment results indicate that ResNet152V2 achieved the best results with a 99.09% accuracy, 99.51% AUC, 99.08% recall, 99.12% precision, and the loss is 0.196 based on the MRI brain images. The novel transfer deep learning model is proposed in Alanazi et al. (2022) to diagnose the brain tumor early by using different subclasses such as glioma, pituitary, and meningioma. To assess their performance for the MRI brain pictures, the Convolutional Neural Network models are created from the ground up. The MRI Brain images are then classified into tumor sub-classes using the different 22-layer, binary-classification (tumor or no tumor) isolated-convolutional neural network model by modifying the neurons' weights using the transfer-learning technique. The transfer learning model is implemented on an unseen MRI brain dataset, and the result indicates that the proposed model provides 96.89% high accuracy.

Authors in Amin et al. (2022) the new model is proposed to detect brain cancer using ensemble transfer learning and Quantum Variational classifiers (QVR). The in-depth features are extracted by the inceptionv3 model in which the score vector is obtained by softmax and used the QVR for discrimination among pituitary tumor, no tumor, meningioma, and glioma. The research is implemented on three different datasets such as 2020-BRATS, local images, and Kaggle, and the proposed model achieved more than 90%detection score. The NeuroXAI framework is proposed in Zeineldin et al. (2022) to explain deep learning networks for increasing medical expert trust. The proposed framework implements the seven different methods such as vanilla gradient, guided back-propagation, integrated gradients, guided integrated gradients, Smooth-Grad, Grad-CAM, and guided Grad-CAM for providing the maps of visualization that help in creating the transparent deep learning model. The framework is implemented on the BraTS 2019 dataset and achieved 98.62% accuracy.

The automated Ultra-Light Brain Tumor Detection (UL-BTD) system is proposed in Qureshi et al. (2022) that is based on the new Ultra-Light Learning Architecture (UL-DLA) for the in-depth features, merged with the textural features that extracted from Gray Level Co-occurrence Matrix (GLCM). It created the Hybrid Feature Space (HFC) for detecting the brain tumor using a support vector machine. The proposed system is implemented on a T1-weighted MRI dataset and achieved the 99.23% average detection rate, and 0.99% is the F1 measure. Different experiments are performed in Senan et al. (2022) to diagnose the brain tumor by combining machine learning and deep learning techniques. For identifying and categorizing brain cancers, support vector machine techniques are combined with ResNEt-18 and AlexNet. A brain tumor MRI images are enlarged using the average filter technique, and deep learning techniques are used to extract the essential features using deep convolutional layers. The extracted features are classified by using SVM and Softmax. The experiment is implemented on an MRI dataset containing 3,060 images and divided into four classes: one is normal, and the other three are tumors. The results indicate that AlexNet with SVM showed the best results with a 95.10% accuracy, 98.50% specificity, and 95.25% sensitivity. The detailed review of CNN architectures is presented in Alsaif et al. (2022) and provides the characteristics of different models such as VGG, ResNet, and AlexNet. The method based on CNN and data augmentation is applied to the MRI dataset to detect the brain tumor, and the result showed that the VGG model achieved a high value with a 0.93% accuracy,0.93% F1-score, 0.94% precision, and 0.93% recall.

Some gaps can be utilized to recognize and detect brain tumors, including ensemble classifiers, machine learning methods, and numerous datasets used to test the generalizability of methods like BRATS, T1-weighted MRI dataset, and deep learning model to categorize the MRI brain pictures. Table 1 summarize the existing literature work. It can be noticed that there exists some work on brain tumor detection but the lack in performance.

**Table 1:** Summery of existing literature work.

**References**	**Focus**	**Technique**	**Limitation**
Alsaif et al. (2022)	Brain tumor	CNN	Low performance
Rehman et al. (2020)	Brain tumor	AdaBoost1 and RUSBoost	Low performance
Salama and Shokry (2022)	Brain tumor	CVG	Low performance

## 3. Proposed methodology

**Figure 1:**
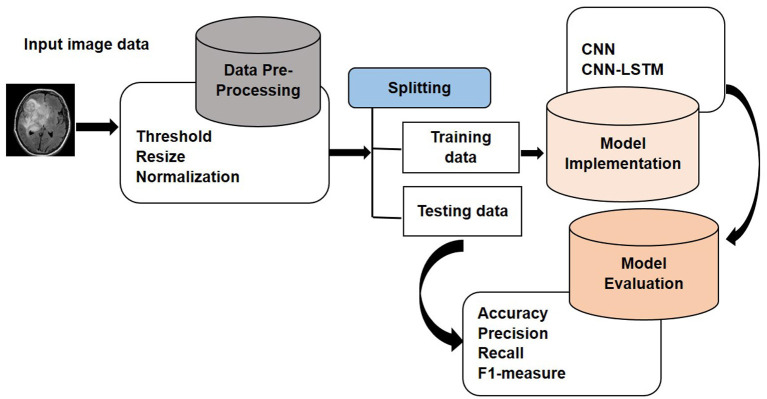
Proposed approach.

In this section, the details of the methods and algorithms of the proposed approach are explained that are used for the classification of MRI Brain images. To validate the proposed approach, accuracy, precision, recall, and F1-measure are calculated as a part of a controlled experiment. Python language is used for conducting a controlled experiment. In an experiment, the impact of the independent variable on the dependent variable is investigated and changed. The accuracy of the suggested model is the dependent variable in this study, while independent variables are employed to examine their effects. The proposed model has four different steps in the first step selects the dataset. In the second step, data pre-processing is performed, which consists of different steps such as thresholding, extreme point calculation, and bicubic interpolation. In the third step, data extraction is performed, and in the last step, the experiment is performed where deep learning technique CNN and CNN-based hybrid deep learning model CNN-LSTM is applied to detect the brain tumor. The proposed technique is presented in Figure 1.

### 3.1. Experimental dataset

**Figure 2:**
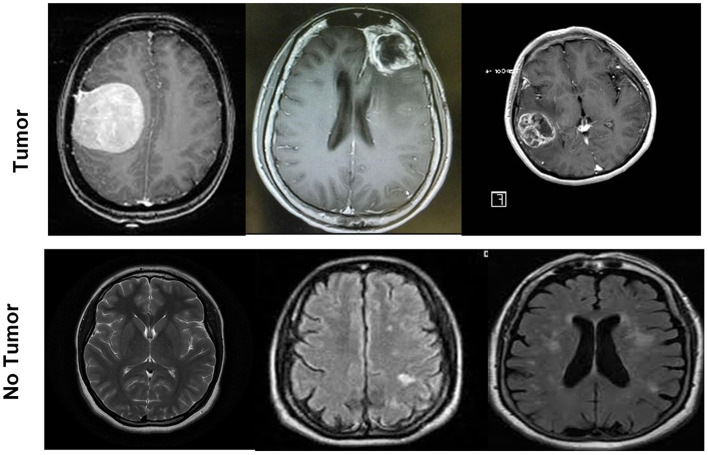
MRI brain images samples for two classes tumor and no tumor.

The system's performance is assessed on the MRI brain tumor dataset. The dataset is gathered from Kaggle, which is the publically available database. Kaggle allows the user to find and publish different datasets, work with different machine and deep learning publishers and data scientists, and design and explore the models in different data science environment (Dahiwade et al., 2019). The dataset consists of a total number of 253 images. The dataset is arranged into one folder (BrainTumorImages) and consists of two sub-folders, one is no tumor with 98 images, and the other is a tumor folder with 155 images. MRI brain image classification is represented in Figure 2.

### 3.2. Data preprocessing

**Figure 3:**
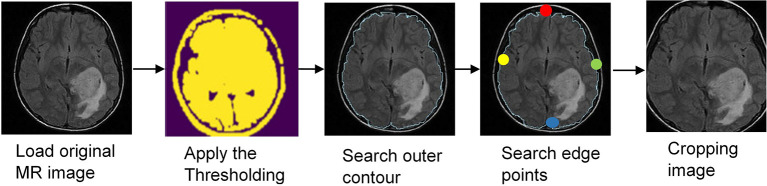
Data preprocessing steps.

In this section, data preprocessing is performed to remove the undesired data in noise form that decreases the model's performance. The unwanted areas and spaces are present in every MRI brain dataset image. Therefore, cropping the images is crucial to remove extraneous space and utilize only the relevant data. This research uses the cropping technique in Dahiwade et al. (2019) that uses the extreme point calculation. The steps to crop the MR images using the extreme point calculation are presented in Figure 3. In the first step for preprocessing, load the actual MR image, convert the images into grayscale and blur it slightly and then apply the thresholding to the magnetic resonance image for converting these images into binary images. The erosion and dilation operations are performed to remove any little noise regions in the images. Afterward, select the most significant contour from threshold images and compute four different extreme points (extreme left, extreme right, extreme bottom, and extreme top). In the last step, crop the image using the collected information from extreme points and contours. By using the bicubic interpolation, crop the MR tumor images. Bicubic interpolation is preferred because it can create a smoother curve compared to other interpolation techniques like bilinear interpolation, and it is the best option for MRI brain tumor images since there is more noise at the edge. In the MRI images dataset, the images are of different sizes, heights, and widths. So it is necessary to resize the images into equal height and width to achieve the best results. In this study, the image is resized to 224 *X* 224 for uniformity. After that, all images are encoded between 0 and 255, and finally, images are normalized.

### 3.3. Feature extraction

After preprocessing the data, the MR image dataset is trained. Features are extracted that represent each tumor. The models based on CNN extract the significant features without any human supervision. The advantage of deep learning decides how to use convolutional filters for extracting the features from the training dataset. In this research, the deep learning models CNN and CNN-LSTM are implemented to classify brain tumor types and extract the in-depth features from an image. The extracted features are then fed to the deep learning models, including a convolutional neural network with a fully connected (FC) layer (Kang et al., 2021).

### 3.4. Convolutional neural network

Convolutional Neural Network is the deep neural network class that utilizes the different convolutional layers to filter inputs for helpful data. The Convolutional filters are applied to the input through the convolutional layers of CNN to compute the output of the neurons connected to specific regions in the input. It helps to extract the temporal and spatial features from an image (Goyal et al., 2019).

**Figure 4:**
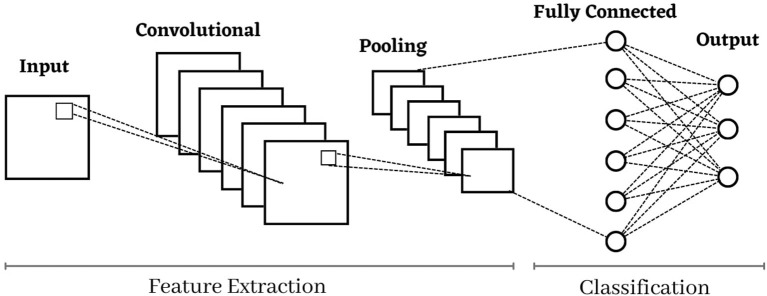
Architecture of convolutional neural network.

The CNN model contains three significant layers a convolutional layer, a max-pooling layer, and a fully connected layer. The convolutional layer contains three significant parameters: pitch, padding, and filter size. Many filters in each layer are used for in-depth feature extraction. According to stride, the filters move within the images. The stride size is either one or two; if the value is more significant than two, CNN performance deteriorates. The zero padding is necessary for retaining the structural assessment when the filter does not shield all of the input images in the convolutional layer. The goal of each convolutional layer is to perform a particular task; for example, the edges of the lesions are highlighted in the first layer, the second layer is used to extract the features of complex geometrics, and in the third layer, the lesions shapes and colors are highlighted. In the feature map, the ReLU layer passes the positive values while negative values are suppressed, turning them into zero (Senan et al., 2022). The max-pooling layer is used to downsample or decrease the dimensionality of the extracted features. The max and average are the two popular methods of the max-pooling layer. The fully connected layer with the 512 unit is used to classify the image into different classes (Goyal et al., 2019; Kang et al., 2021). For feature map normalization, the batch normalization layer is used. These layers accelerate the training and network regulation. The dropout layer is employed in some issues and is highly beneficial to overcome the over-fitting issues with networks (Balaha et al., 2021). The CNN architecture is presented in Figure 4.

### 3.5. Long short term memory

The long short-term memory is the improved version of the Recurrent Neural Network (RNN). Long-term memory suggests the memory blocks rather than traditional units of RNN to solve the problem of exploding gradient and vanishing (Amin et al., 2020). The main difference between LSTM and RNN is that LSTM adds the cell state for saving the long-term states. An LSTM network can recall and link data from the past to the information acquired in the present (Chen, 2016). The long short-term memory is designed with three different gates, the input gate, forget gate, and the output gate. The current input is represented by *u*_*t*_ the new and the previous states of the cell are represented by *s*_*t*_ and s_*t*−1_ respectively, and the current and the previous output is denoted by v_*t*_ and v_*t*−1_ respectively. The rules for the input gate of the LSTM are given in the below equations.

In Equation (1) pass the previous output v_*t*−1_ and u_*t*_ by the sigmoid layer to determine which part of the information has to be added by using the *J*_*t*_ to take the input information while *a*_*i*_ represents the input gate.


(1) $$\begin{array}{r} J_{t}=\sigma \left(W_{i}.\left[v_{t-1},u_{t}\right]+a_{i}\right) \end{array}$$

The Equation (2) is used to get the updated information S_*t*_ after passing the previous information v_*t*−1_ and current information u_*t*_ by *tanh* layer using the input gate *a*_*i*_. The current state of information s_*t*_, and information of long-term memory S_*t*−1_ into *S*_*t*_ are integrated in Equation (3), where sigmoid output is referred by W_*i*_ and S_*t*_is referred the *tanh* output. The weight metrics are represented by W_*i*_, and the input gate of the LSTM is represented by a_*i*_. The forget gate of the LSTM then permits the selective transmission of the information by using the dot product of input information *J*_*t*_ and the current state of information s_*t*_ and sigmoid layer.


(2) $$\begin{array}{r} s_{t}=tanh\left(W_{i}.\left[v_{t-1},u_{t}\right]+a_{i}\right) \end{array}$$

(3) $$\begin{array}{r} S_{t}=h_{t}\left(S_{t-1}\right)+j_{t}s_{t} \end{array}$$

With the specific probability, it is decided whether to delete the related data by the last cell by using Equation (4), where W_*f*_ represents the weight matrix, offset is a_*f*_ and sigmoid function is σ.


(4) $$\begin{array}{r} b_{t}=\sigma \left(W_{f}.\left[v_{t-1},u_{t}\right]+a_{f}\right) \end{array}$$

The inputs in Equations (5) and (6) determine that the output gate *O*_*t*_ of the LSTM describes the states that are needed for the continuation through the previous and current output v_*t*−1_ and u_*t*_ respectively. The final output *V*_*t*_ is multiplied and acquired through the decision vector of the state that transfers the new data S_*t*_by the *tanh* layer.


(5) $$\begin{array}{r} O_{t}=\sigma \left(W_{o}.\left[v_{t-1},u_{t}\right]+a_{o}\right) \end{array}$$

(6) $$\begin{array}{r} V_{t}=O_{t}tanh\left(S_{t}\right) \end{array}$$

Where the W_*o*_ is the weighted matrix of the output gate a_*o*_ is the bias of LSTM of output gate the (Islam et al., 2020).

**Figure 5:**
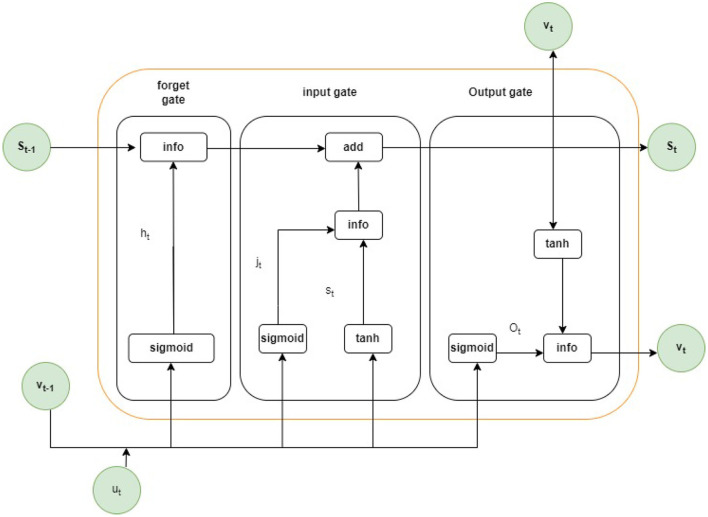
Architecture of LSTM model.

The architecture of LSTM model is presented in Figure 5. The model has three gates: the input, forget, and output. The first gate is forget gate (*h*_*t*_) that takes the previous output v_*t*−1_ and current input *u*_*t*_ form previous state s_*t*−1_ with sigmoid function σ. The input gate add the previous information and take input information *J*_*t*_ by *tanh* layer and sigmoid function σ. To get the output, the output gate is used where the information comes from the input gate, and the output gate computes all information by *tanh* layer and uses the sigmoid function σ and provides the current state where the output is stored.

### 3.6. Proposed hybrid CNN-LSTM model

In this research, the combined model is developed to detect the brain tumor automatically. The architecture's structure is created by combining the CNN and networks of LSTM, where CNN extracts the features, and as a classifier, LSTM is used.

**Figure 6:**
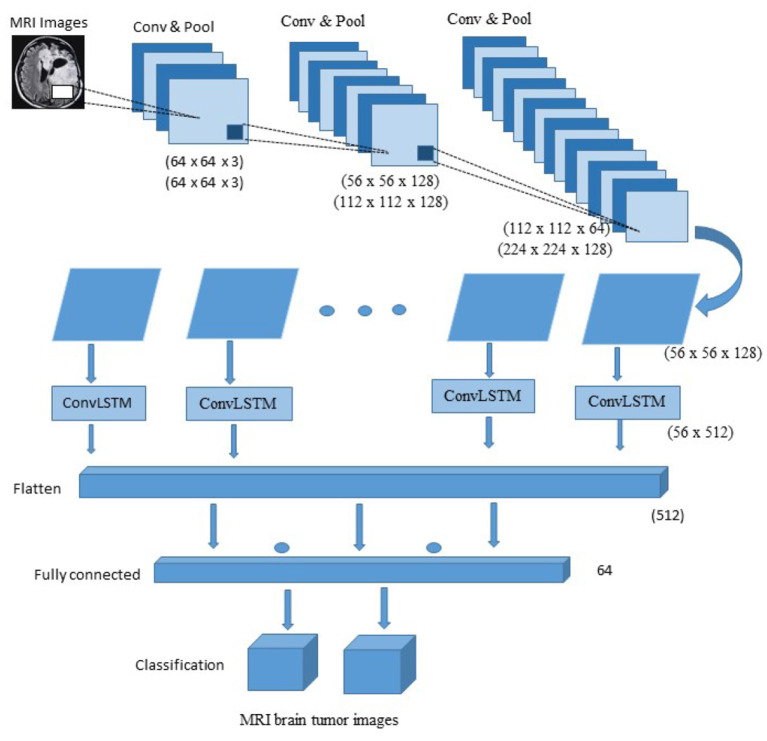
Architecture of proposed hybrid CNN-LSTM model.

Figure 6 presents the proposed hybrid model for brain tumor detection. The network has 16 layers, three convolutional layers, seven max-pooling layers, four ConvLSTM layers, one fully connected layer, and the last one is the output layer connected with the softmax function. The proposed model first uses the three convolutional layers as CNN layers with three max-pooling layers and then uses the four ConvLSTM with four max-pooling layers. Each ConvLSTM and the convolutional block are integrated with a 2D convolutional neural network, one max-pooling layer, and a dropout layer with a 25% rate. The convolutional layer with a kernel size of 3 x 3 is used to extract the features from MRI brain images activated through the function of Relu. The max-pooling layer with kernel size 2 x 2 reduces the input image dimensionality. The convolutional layer and max-pooling layers are used with different input sizes such as (64,64,3), (56, 56,128), (112, 112, 128), (112, 112, 64) and (224, 224, 128) for less shrinking of the images. Fewer shrinkage results in less distortion of the image's internal characteristics and patterns. After the convolutional block, the output shape is found as the (56, 56, 128) given in the ellipsis form in Figure 6. Then using the reshaping method to resize the images into equal height and width to achieve the best results, after the reshaping method, the size of the ConvLSTM layer becomes (56,512) used for sequence prediction. After reshaping the ConvLSTM layer, the size of the fully connected layer and the output layer has 512 and 64, respectively. In the architecture's last part, The ConvLSTM layer is used for extracting the information about time. The architecture categorizes the MRI brain tumor image, either tumor or non-tumor, by a fully connected layer after looking at the characteristics of the time.

In Table 2 summary of the proposed architecture CNN-LSTM is provided. The layers types, kernel size, and input size are provided in detail. The convolutional2D layer has the 3 x 3 size and different input sizes such as (64 x64 x 3) and (56 x 56 x 128). The maxpooling2D layer has the 2 x 2 kernel size with (64 x 64 x 3), (56 x 56 x 128), (112 x 112 x 64), (112 x 112 x 128) and (14 x 14 x 128) input size. The ConvLSTM2D layer has kernel size 3 x 3 with different input sizes such as (112 x 112 x 64), (112 x 112 x 128) and (224 x 224 x 128). The fully connected layer and the output layer have the input sizes 512 and 64, respectively. The convolutional and max-pooling layers are employed for reduced image compression with varied input sizes. Less contraction leads to less distortion of the internal traits and patterns of the image. By a fully connected layer, the architecture sorts the MRI brain tumor image, either tumor or not tumor, after analyzing the characteristics of the time. The Adam optimizer is used for training the proposed model with a batch size of 32, the number of epochs is 100, and cross-entropy is used for losses and the metrics for accuracy.

**Table 2:** The CNN-LSTM network summary.

**Layers**	**Types**	**Kernel size**	**Input size**
1	Convolutional2D	3 x 3	64 x 64 x 3
2	MaxPooling2D	2 x 2	64 x 64 x 3
3	Convolutional2D	3 x 3	56 x 56 x 128
4	MaxPooling2D	2 x 2	56 x 56 x 128
5	Convolutional2D	3 x 3	112 x 112 x 64
6	MaxPooling2D	2 x 2	112 x 112 x 64
7	ConvLSTM2D	3 x 3	112 x 112 x 64
8	MaxPooling2D	2 x 2	112 x 112 x 128
9	ConvLSTM2D	3 x 3	112 x 112 x 128
10	MaxPooling2D	2 x 2	14 x 14 x 128
11	ConvLSTM2D	3 x 3	112 x 112 x 128
12	MaxPooling2D	2 x 2	112 x 112 x 128
13	ConvLSTM2D	3 x 3	224 x 224 x 128
14	MaxPooling2D	3 x 3	224 x 224 x 128
15	FC	–	512
16	Output	–	64

The steps in the proposed model CNN-LSTM are given in Algorithm 1. The image from the MR brain tumor dataset is taken as input. Image preprocessing is performed by resizing the image to a size of 224 x 224 and cropping the images through the extreme point calculation method. Split the data into the parts of training (70%) and validation (30%). After this, classify and extract the features through a convolutional neural network and hybrid deep learning model CNN-LSTM with 100 epochs and 32 batch sizes. Calculate the loss through cross entropy and apply optimization and fitness functions to validate and train the model. Calculate the performance metrics and obtain the result.

**Algorithm 1:** Pseudo code of Proposed CNN-LSTM model

1:	Dataset ← X, Y = {y_1_, y_2_, y_3_, …, y_*n*_}
2:	Performs image pre-processing
3:	**Image** = cv2.resize (224 x 224), resize the image.
4:	Computing **Newimage** = image (extTop [1]: extBot [1]: extLeft [0]: extRight [0]), Cropping the image using extreme point calculation.
5:	Splitting the dataset into validation and training parts. Thirty percent for validation and 70% for training.
6:	**CNN-LSTM** ← Classifying and extracting the feature through deep learning models.
7:	F = (f_1_, f_2_,f_3_, …, f_*n*_) map the feature extraction vector into high dimensional space.
8:	**for** every epoch in the number of epochs **do**
9:	**for** every batch in the batch-size **do**
10:	x = model (F);
11:	Loss = cross_entropy (X, x), Calculate the loss
12:	Optimization and fitting function applied for validation and training of the model
13:	Compute the validation metrics: precision, accuracy, F1-measure, and recall
14:	**end for**
15:	**end for**
16:	**return** Results

## 4. Result and discussion

In this section, analyze the proposed model performance. The proposed model is evaluated on different parameters. These parameters are carried out to determine whether the proposed model is better than the previous methods and whether it is appropriate for brain tumor detection or not. The proposed model is implemented on an MRI brain image dataset, and a deep learning model is applied to this dataset.

### 4.1. Evaluation metrics

Different evaluation metrics are used for the prediction and classification challenges, such as accuracy, precision, recall, and F1-measure (Rehman et al., 2022a). The effectiveness of the proposed model is assessed using the following evaluation metrics.

*Accuracy:* To measure the accuracy of the proposed model, compute the ratio of the false positive, true positive, true negative, and false negative. The Equation (7) represent the accuracy estimate.


(7) $$\begin{array}{r} Accuracy=\frac{TP+TN}{TP+TN+FP+FN} \end{array}$$

*Precision:* The ratio of real positives to all positives overall (both false and true) in the data. Additionally referred to as a high predicted value. The precision rate is represented in Equation (8).


(8) $$\begin{array}{r} Precision=\frac{TP}{TP+FP} \end{array}$$

*Recall:* The ratio of the true positives in the data to true positives and false negatives is often referred to as sensitivity, the chance of detection, and the rate of a true positive. The recall rate is represented in Equation (9).


(9) $$\begin{array}{r} Recall=\frac{TP}{TN+FN} \end{array}$$

*F1-measure:* The weighted average of the precision and recall is known as the F1 measure. The Equation (10) represents the value of F1-measure.


(10) $$\begin{array}{r} F1-measure=2\times \frac{Precision+Recall}{Precision+Recall} \end{array}$$

### 4.2. Experimental analysis and result

**Figure 7:**
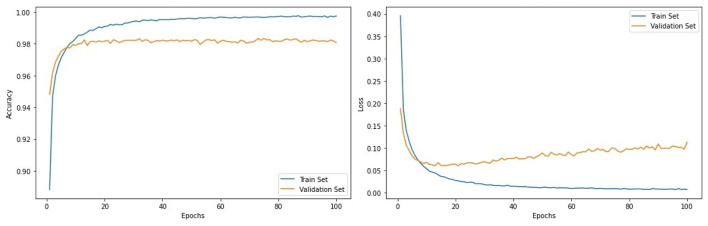
Evaluation metrics of CNN architecture.

The proposed technique is applied to deep learning models CNN and CNN-LSTM. Figure 7 represents the performance evaluation of the convolutional neural network with training and validation loss and accuracy. At every 100 epochs, the training accuracy is 99.4%, and validation accuracy is 98.3%. Similarly, the training loss is 0.007, and 0.113 is the validation loss.

**Figure 8:**
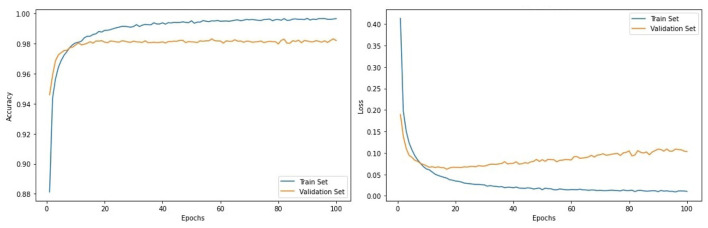
Evaluation metrics of CNN-LSTM architecture.

The performance evaluation of the CNN-LSTM with training and validation loss and accuracy is represented in Figure 8. At every 100 epochs, the training and validation accuracy is 99.8 and 98.5%, respectively. Similarly, the training loss is 0.010, and 0.103 is the validation loss.

Table 3 provides the results of proposed technique. The CNN achieved the accuracy is 98.6%, the precision is 98.5%, a recall is 98.6%, and F1-measure is 98.4%. The hybrid deep learning model CNN-LSTM achieved an accuracy of 99.1%, a precision of 98.8%, a recall of 98.9%, and an F1-measure of 99.0%.

**Table 3:** Performance of CNN and Hybrid CNN-LSTM model.

**Model**	**Accuracy**	**Precision %**	**Recall**	**F1-Measure**
CNN	98.6	98.5	98.6	98.4
CNN-LSTM	99.1	98.8	98.9	99.0

**Figure 9:**
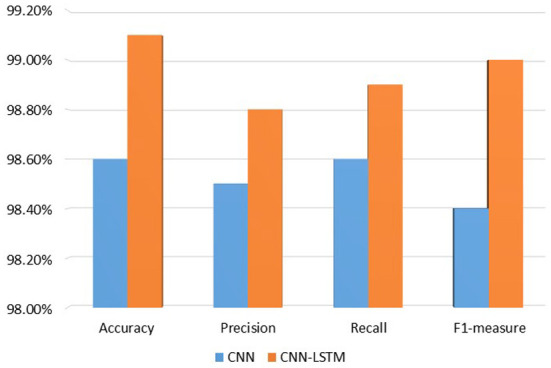
Performance of proposed model.

Figure 9 graphically represents the performance of the proposed model. The proposed model CNN-LSTM performs well compared to the deep learning technique CNN in terms of accuracy 99.1%, precision 98.8%, recall 98.8%, and F1-measure 99.0%.

### 4.3. Comparative analysis

The comparison of the proposed model with previous techniques is provided in Table 4. The same preprocessing steps are performed in the previous techniques as the preprocessing steps for the proposed model.

**Table 4:** Comparison with previous techniques.

**References**	**Techniques**	**Accuracy**	**Precision%**	**Recall**	**F1-measure**
Alsaif et al. (2022)	CNN	93.0	94.0	93.0	93.0
Rehman et al. (2020)	AdaBoost1 and RUSBoost	98.0	88.0	NA	NA
Salama and Shokry (2022)	CVG	97.0	96.9	96.0	96.9
Proposed approach	CNN-LSTM	99.1	98.8	98.9	99.0

Authors in Alsaif et al. (2022) provides the result in term of accuracy 93%, precision 94%, recall 93% and F1-measure 93%. Authors in Rehman et al. (2020) achieved the result in terms of accuracy 98% and precision 88%. Authors in Salama and Shokry (2022) achieved the results in terms of accuracy 97%, precision 96.88%, recall 96%, and F1-measure of 96.88%.

**Figure 10:**
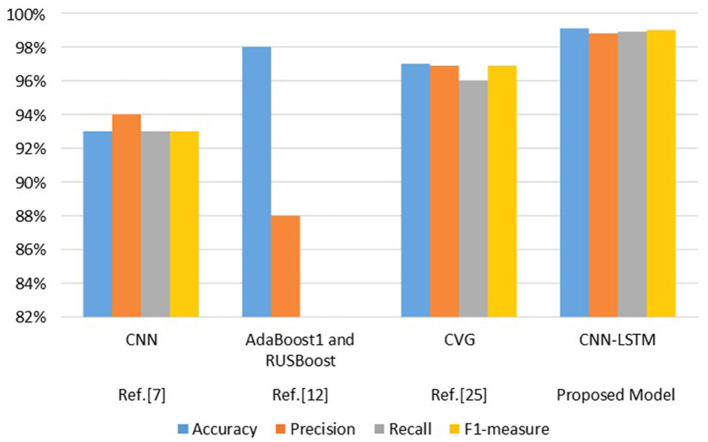
Comparison of proposed model with existing techniques.

In Figure 10 the comparison of the proposed model with the existing one is graphically represented. It seems that the proposed model is performing better than previous techniques.

## 5. Conclusion

Detecting a brain tumor is complicated because of the brain's complex structure. Every organ in the body has a function that is controlled by the brain. Automatic initial stage brain tumor categorization using deep learning and machine learning techniques plays a crucial part. These systems enable prompt diagnosis and raise the likelihood of survival for patients. Additionally, these methods support specialists and radiologists in their decision-making regarding diagnoses and plans for treatment. This study proposed the CNN-based hybrid deep learning model CNN-LSTM to classify the brain tumors using the MR brain tumor images dataset; firstly, the image dataset is by thresholding, extreme point calculation, and bicubic interpolation. Secondly, the proposed model uses the convolutional neural network for extracting the features in the form of cropped images. Four metrics, accuracy, precision, recall, and F1-measure, are used to evaluate the model's performance. The proposed model provides the best result by achieving 99.1% accuracy, precision is 98.8%, recall is 98.9%, and F1-measure is 99.0%. The results showed that the proposed model is best for detecting the MR brain images. The future work would be to investigate the performance of the proposed approach on multi-class MR brain tumor images problem and use different datasets such as Brast2022 and T-weighted to enhance the performance of the proposed model.

## Data availability statement

The original contributions presented in the study are included in the article/supplementary material, further inquiries can be directed to the corresponding authors.

## Author contributions

All authors listed have made a substantial, direct, and intellectual contribution to the work and approved it for publication.

## Funding

The research is supported by Qatar National Library and Qatar university internal grant IRCC-2021-010.

## Conflict of interest

The authors declare that the research was conducted in the absence of any commercial or financial relationships that could be construed as a potential conflict of interest.

## Publisher's note

All claims expressed in this article are solely those of the authors and do not necessarily represent those of their affiliated organizations, or those of the publisher, the editors and the reviewers. Any product that may be evaluated in this article, or claim that may be made by its manufacturer, is not guaranteed or endorsed by the publisher.
